# Lownds County (Miss.) Medical Association

**Published:** 1871-12

**Authors:** W. L. Lipscomb, J. W. M. Shattuck

**Affiliations:** President; Secretary


### LOWNDES COUNTY (MISS) MEDICAL ASSOCIATION.

The Association met, pursuant to adjournment, at Columbus, July 22d, 1871. The Rev. Dr. Lipscomb, in the Chair. The minutes of the preceding meeting were read and adopted.

Dr. Brownrigg reported a case in which quinine, in any form, produced unusual effects. Such as an eruption similating Unticaria or Erysipelas, with icy coldness of the extremities and edema, together with dangerous asphyxia, requiring large doses of alcoholic stimulants. He gave three gobletfuls of whisky in less than one hour, without producing any intoxicating effects.

Drs. Lipscomb and Vaughan also, reported cases in which quinine produced, or was followed by Unticaria, but they had not seen such poisonous results from quinine.

The President reported a case in which quinine, even in very small doses, always produced hæmaturia. Similar cases were reported by several other members.

Dr. Maxwell reported a case of spasms in a child 18 months old, who had, at least, forty spasms before he saw the patient. The parents, who were very intelligent, had tried all the usual remedies without any apparent benefit, the Doctor had to do something, and he decided to use quinine. He gave 5 grs., and in one hour, gave 2 grs. more. The child had no more spasms, only slight twitching of the muscles. The case recovered rapidly and perfectly.

This case was discussed by Drs. Lipscomb, Lyon, Franklin and Vaughan, they considered the treatment good, the quinine preventing a return of the spasms, by acting as a nervous sedative, or by counteracting nervous depression.

Dr. Brownrigg suggested the efficacy of fly-blisters to the epigastrium and abdomen, in spasms resulting from gastric or intestinal irritation. Dr. Vaughan advocated the use of blisters in spasms, but would apply them to the spine instead of the epigastrium, as he believed they produced a much better effect.

The President remarked, that he did not believe there could be a well-marked spasm without the brain being effected; and that, in a case of spasms, if, after the first or second convulsion, the little patient became rational and entirely sensible, he would recover.

Dr. Maxwell inquired of the Chair, whether the use of opiates in spasms, was good practice or admissable? The President replied that it was, but to know when to use them was a nice point.

Dr. Maxwell remarked, that he had discarded them in these cases, and had succeeded much better than when he used them.

Several members thought that we often attached too much importance to convulsions in children.

Dr. Vaughan said, he knew a boy who was raised on spasms, having had several hundred. This closed the discussion, for this meeting.

Dr. Lyon was appointed Essayist for the regular meeting in August, 1872.

The Association adjourned to meet August 26th, 1872.

W. L. LIPSCOMB, M. D., *President.*

J. W. M. SHATTUCK, M. D., *Secretary.*

